# Oxygen Migration Pathways in Layered LnBaCo_2_O_6-δ_ (Ln = La – Y) Perovskites

**DOI:** 10.1021/jacsau.4c00049

**Published:** 2024-04-02

**Authors:** Fabian Hesse, Ivan da Silva, Jan-Willem G. Bos

**Affiliations:** † Institute of Chemical Sciences, School of Engineering and Physical Sciences, Heriot-Watt University, Edinburgh EH14 4AS, U.K.; ‡ ISIS Facility, Rutherford Appleton Laboratory, Harwell Oxford, Didcot OX11 0QX, U.K.; § EaStCHEM School of Chemistry, University of St Andrews, North Haugh, St Andrews KY16 9ST, U.K.

**Keywords:** BVSE calculations, oxygen vacancy ordering, cobalt oxide perovskites, layered double perovskite, mixed ionic electronic conductor, neutron powder diffraction

## Abstract

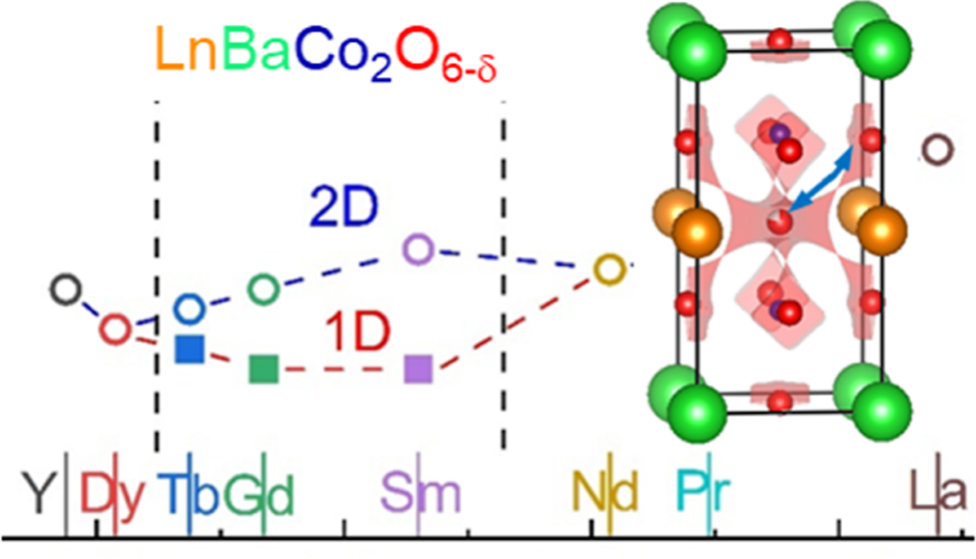

Layered LnBaCo_2_O_6-δ_ perovskites are important mixed ionic-electronic conductors, exhibiting outstanding catalytic properties for the oxygen evolution/reduction reaction. These phases exhibit considerable structural complexity, in particular, near room temperature, where a number of oxygen vacancy ordered superstructures are found. This study uses bond valence site energy calculations to demonstrate the key underlying structural features that favor facile ionic migration. BVSE calculations show that the 1D vacancy ordering for Ln = Sm–Tb could be beneficial at low temperatures as new pathways with reduced barriers emerge. By contrast, the 2D vacancy ordering for Ln = Dy and Y is not beneficial for ionic transport with the basic layered parent material having lower migration barriers. Overall, the key criterion for low migration barriers is an expanded *ab* plane, supported by Ba, coupled to a small Ln size. Hence, Ln = Y should be the best composition, but this is stymied by the low temperature 2D vacancy ordering and moderate temperature stability. The evolution of the oxygen cycling capability of these materials is also reported.

## Introduction

Layered, A-site ordered, LnBaCo_2_O_6-δ_ (Ln = rare earth) double perovskites are multifunctional energy materials with promising performance as cathodes in solid oxide fuel cells,^1−9^ as oxygen separation membranes,^10,11^ and as catalysts for the oxygen evolution reaction in electrolysis.^12−14^ The outstanding catalytic properties derive from the mixed ionic-electronic conductivity, which is advantageous as it allows chemical reaction to occur over the entire surface and not only at triple phase boundaries. The good ionic conduction in these materials has been linked to the high vacancy concentration (0 ≤ δ ≤ 1), low migration barriers for bulk oxide ion transport, and high oxygen surface exchange coefficients.^1,3−5,15,16^

Despite the large amount of work on individual LnBaCo_2_O_6-δ_ compositions, a comparison of migration barriers for oxide ion transport across all structures, i.e., exploring the impact of Ln cations and oxygen vacancy ordering patterns, is lacking. This study addresses this gap in the literature and reports the stability (oxygen cycling capacity) for all stable LnBaCo_2_O_6-δ_ compositions, prepared and analyzed in a consistent manner. Although not included in this work, Ln and oxygen vacancies also influence electrical conduction through Co-oxidation state, and structural factors such as the degree of orbital overlap with oxygen ligands. Typical electrical conductivities for the LnBaCo_2_O_6-δ_ materials are 100–1000 S cm^–1^.^3−5,17^

**Figure 1:**
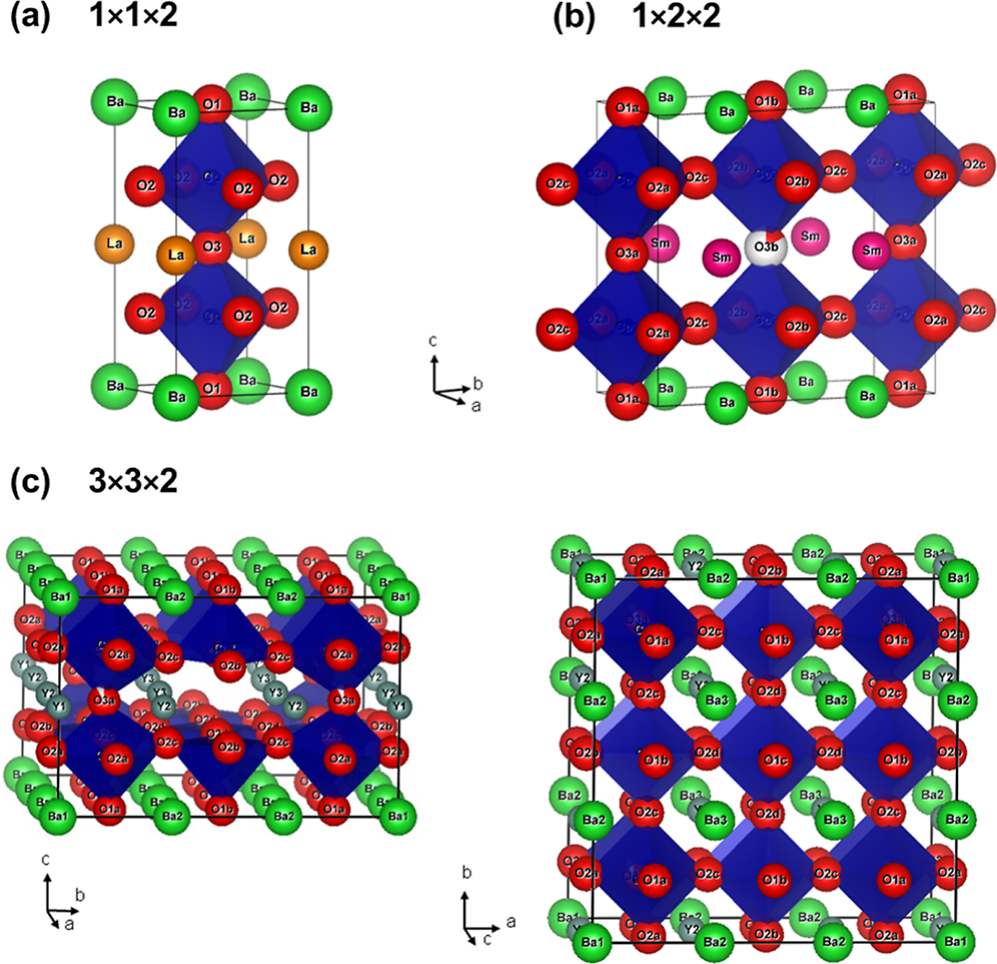
Schematic representation of the reported crystal structures for the layered LnBaO_2_O_6-δ_ perovskites: (a) tetragonal *P*4/*mmm* 1 × 1 × 2 parent structure for LaBaCo_2_O_6_. (b) Orthorhombic *Pmmm* 1 × 2 × 2 superstructure for SmBaCo_2_O_5.59_ with a largely vacant O3b site, leading to 1D vacancy-rich channels running along the *a* direction. (c) Tetragonal *P*4/*mmm* 3 × 3 × 2 superstructure for YBaCo_2_O_5.41_ with empty O3b and O3c sites, leading to two symmetry equivalent perpendicular 1D vacancy channels along the *a*- and *b*-directions.

The crystal structure of the LnBaCo_2_O_6-δ_ perovskites shows a strong dependence on Ln and the oxygen content (δ).^18,19^ The parent structure is tetragonal and has Ln^3+^ and Ba^2+^ cations in alternating layers, leading to a doubling of the simple cubic perovskite unit cell in the *c* direction. This gives an a_p_ × a_p_ × 2a_p_ superstructure, subsequently referred to as 1 × 1 × 2 (Figure 1a). The oxygen content (δ) after synthesis shows a strong dependence on Ln, with greater oxygen deficiency found for smaller Ln ions.^18,19^ This trend is driven by lattice enthalpy, where smaller Ln are more stable with reduced oxygen content.^1^ The oxygen vacancies are largely found in the Ln-O layers in the crystal structure.^18,19^ Two types of oxygen vacancy ordering have been reported: the first is characterized by an orthorhombic 1 × 2 × 2 superstructure with 1D vacancy ordered chains running along the *a* direction in the Ln-O plane (Figure 1b). This occurs near δ = 0.25 and is observed to values δ = 0.55. At higher oxygen vacancy concentrations (δ ≥ 5/9), a different tetragonal 3 × 3 × 2 superstructure occurs (Figure 1c).^18−20^ This is characterized by two perpendicular 1D channels that are depleted of oxygen. After synthesis in air, La, Pr, and Nd typically have the 1 × 1 × 2 structure, Sm, Gd, and Tb have the 1 × 2 × 2 structure, and Dy and Y are found with the 3 × 3 × 2 structure.^18−20^ Oxygen contents below δ = 1 can be reached by topotactic reduction using metal hydrides affording compositions with δ = 1.5 and δ = 1.75,^21,22^ but these are not accessible using regular high temperature routes. Heating tetragonal LaBaCo_2_O_6-δ_ in reducing atmospheres (flowing N_2_) removes oxygen and leads to the observation of the 1 × 2 × 2 structure near 350 °C, before reverting to the tetragonal phase as the oxygen vacancies become disordered.^23^ A similar evolution is observed for TbBaCo_2_O_6-δ_, which has the 1 × 2 × 2 structure after synthesis and converts to the 1 × 1 × 2 structure upon heating and as oxygen is removed.^24^

Table 1 gives an overview of activation barriers for oxide ion conduction from impedance spectroscopy (EIS), isotope exchange and depth profiling (IEDP) and molecular dynamics (MD) and density functional theory (DFT) simulations. Measured values fall between 0.5 and 1.8 eV, showing considerable variation between different techniques, and not showing a clear correlation to Ln. The variation in reported *E*_*a*_ is partly due to the difficulty in measuring ionic conductivity in a mixed conductor material using EIS.^6^ Instead EIS data are collected on symmetric cells that contain an electron blocking but ionic conducting central layer, leading to ambiguity extracting the ionic conductivity of the MIEC. Another reason for the discrepancy between different reports is that oxide-ion transport rates are limited by either the surface-exchange or oxide-ion diffusion coefficient.^5,15,25^ Hence, variations in microstructure between samples can cause differences in EIS and IEDP. Simulations,^26−32^ diffraction,^23,33,34^ and total scattering experiments^35^ have been used to establish the mechanism for ionic conductivity. These studies show that ionic motion is predominantly between the O3 sites in the Ln-O layers and the O2 sites in the Co–O_2_ layers. This leads to a···O2–O3–O2–O3–O2···. “zig-zag” migration pathway (illustrated in Figure 4a). For the tetragonal 1 × 1 × 2 structure, this is isotropic in the *ab* plane, while the orthorhombic 1 × 2 × 2 structure allows different energy barriers for transport along the *a* and *b* axes.^23,27,28^ DFT and MD give a range of migration/activation barriers, clustered near 0.7–1 eV, i.e., comparable to typical EIS and IEDP values, while some MD studies yield much lower values 0.3–0.5 eV (Table 1). 3D transport involving O1 sites in the Ba–O layers carries a very large energy penalty and is strongly disfavored, leading to 2D oxide ion transport at all practical application temperatures.^23,28,31^

**Table 1:** Overview of Reported Activation Energies (*E*_*a*_) for Oxygen Diffusion and Energy Barriers (*E*_*b*_) for Oxygen Migration in LnBaCo_2_O_6-δ_ Double Perovskites^a^

LnBaCo_2_O_6-δ_	*E*_*a*_ (eV)/EIS	*E*_*a*_ (eV)/IEDP	*E*_*b*_ (eV)/MD	*E*_*b*_ (eV)/DFT
La			0.69^26^	
Pr	0.95–1.19^6,49^	0.48–1.02^50−52^	0.27–0.35^30,32^	0.47–0.99^27−29^
Nd	1.19–1.68^53,54^			
Sm	1.23^55^			
Gd	1.21–1.75^54,56,57^	0.48–0.60^15,16^	0.50–0.83^26,31,58,59^	
Y	0.49–0.92^60,61^		0.78^26^	

a *E*_*a*_ values are from EIS and IEDP. *E*_*b*_ values are from MD simulations and DFT calculations.

BVSE calculations are a computationally inexpensive way to calculate migration barriers (*E*_*b*_) for ionic transport directly from the unit cell structure.^36,37^ They use potentials derived from self-consistent bond valence parameters that are available for many ionic pairs.^38,39^ In these calculations, the energy of a tracer ion is calculated for a fine grid of coordinates within the experimentally determined unit cell. This enables energy surfaces to be mapped and barriers to ionic migration to be determined. BVSE has been applied to many inorganic materials and is suited to “translating” structural changes into migration barrier trends.^40−43^ In many cases, good quantitative agreement with more sophisticated approaches, such as DFT and advanced MD simulations, is found. This is, in particular, true when the framework structure does not distort strongly when ions are migrating. Like DFT, BVSE uses a static structure as input but allows for a much finer exploration of the unit cell interior and not only along predefined paths using the nudged elastic band approach. MD is different because it simulates ionic displacements at high temperatures and extracts activation energies (*E*_*a*_) from the Arrhenius dependence of calculated diffusivities. The analysis of migration paths is done via a statistical analysis of the simulated oxygen positions. BVSE is a good complementary approach to DFT and MD that enables the impact of structural changes to be explored in a computationally inexpensive, self-consistent manner.

This work presents a comparison of ionic migration pathways for all stable LnBaCo_2_O_6-δ_ materials. For the smallest Ln possible: Y, variable temperature neutron powder diffraction (NPD) and BVSE calculations were used to probe stability and ion migration of the 3 × 3 × 2 vacancy ordered superstructure.

## Results and Discussion

### Structural Characterization of the LnBaCo_2_O_6-δ_ Series

**Figure 2:**
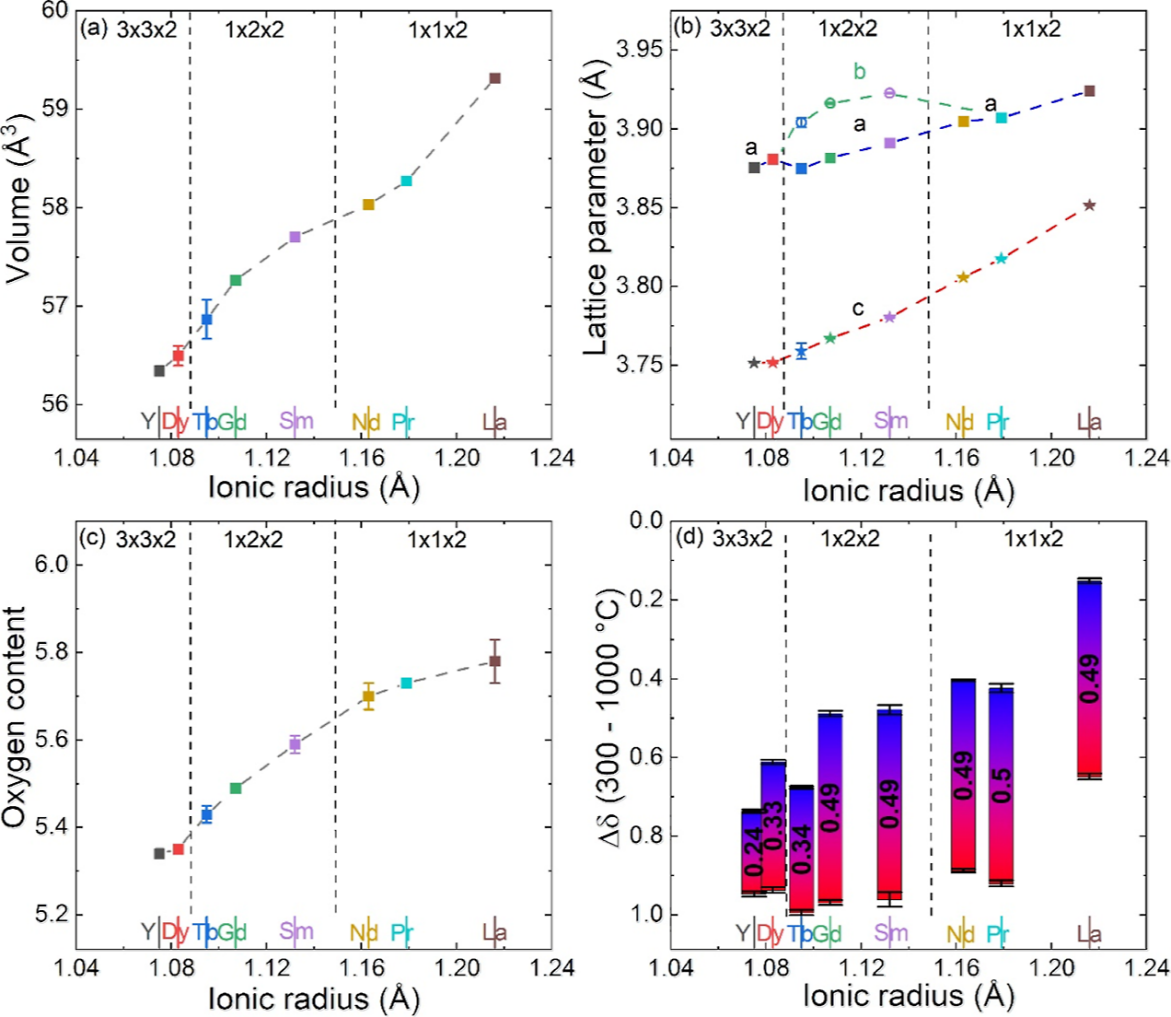
Structural and oxygen cycling trends for the layered LnBaCo_2_O_6-δ_ (Ln = La, Pr, Nd, Sm, Gd, Tb, Dy, and Y) perovskites plotted against ionic radius of lanthanides: (a) normalized unit cell volume from Rietveld analysis of laboratory XRD data; (b) normalized lattice parameters *a*, *b*, and *c*; (c) oxygen content obtained by iodometric titration; and (d) changes in oxygen content between 300 °C (blue) and 1000 °C (red) under flowing N_2_ obtained by TGA.

Analysis of X-ray powder diffraction data (Figures S1, S2) confirmed that the materials had good purity and that they formed with the expected crystal structures.^18−20^ These are the basic layered 1 × 1 × 2 tetragonal structure (La, Pr, and Nd), the 1 × 2 × 2 orthorhombic (Sm, Gd, and Tb), and 3 × 3 × 2 tetragonal (Dy and Y) superstructures. The evolution of the cell volume and lattice parameters is summarized in Figure 2a,b and Table 2. These values have all been normalized to the basic 1 × 1 × 1 unit cell to facilitate comparison. The cell metrics are characterized by a global reduction with decreasing Ln radius. Superposed on this is an increased *b* axis parameter for samples that have the 1 × 2 × 2 superstructure. This leads to an increased cell volume compared with the globally decreasing trend. The overall contraction is driven by the reduction of the *c* axis (∼0.1 Å) and is a consequence of the decreasing size of the Ln^3+^ cations. The basal plane dimensions show a smaller contraction (∼0.05 Å), reflecting the competition between the decreasing Ln^3+^ size and the presence of large Ba^2+^ ions. These trends are consistent with the published literature.^18^ The evolution of the oxygen content from chemical titration is listed in Figure 2c. These data confirm literature trends and reveal a gradual reduction in oxygen content with decreasing Ln^3+^ radius with the boundaries between the 1 × 1 × 2, 1 × 2 × 2, and 3 × 3 × 2 structures occurring at δ ∼ 0.35 and δ ∼ 0.62, respectively. Note that layered LaBaCo_2_O_6-δ_ needs to be prepared under flowing Ar,^23^ and hence has a lower oxygen content than expected based on the trend of the other Ln, which are all prepared in air.

**Table 2:** Overview of Structural and Oxygen Cycling Parameters for the Investigated LnBaCo_2_O_6-δ_ Compositions^a^

Ln	ionic radius (Å)	space group	lattice parameters (Å)			volume (Å^3^)	unit cell	oxygen content	Δoxygen content
			*a*	*b*	*c*				
La	1.216	P*4/*mmm	3.9243 (3)		7.7032 (2)	118.63 (3)	a_p_ ×a_p_ × 2a_p_	5.78 (5)	0.49 (3)
Pr	1.179	P*4/*mmm	3.9069 (1)		7.6355 (2)	116.546 (8)	a_p_ × a_p_ × 2a_p_	5.73 (1)	0.50 (2)
Nd	1.163	P*4/*mmm	3.90481 (9)		7.6118 (1)	116.061 (7)	a_p_ × a_p_ × 2a_p_	5.70 (3)	0.50 (3)
Sm	1.132	*Pmmm*	3.8911 (2)	7.8455 (4)	7.5610 (4)	230.82 (3)	a_p_ × 2a_p_ × 2a_p_	5.59 (2)	0.49 (1)
Gd	1.107	*Pmmm*	3.8815 (3)	7.8324 (5)	7.5345 (5)	229.06 (4)	a_p_ × 2a_p_ × 2a_p_	5.49 (1)	0.49 (3)
Tb	1.095	*Pmmm*	3.875 (2)	7.808 (3)	7.518 (3)	227.5 (3)	a_p_ × 2a_p_ × 2a_p_	5.43 (2)	0.34 (5)
Dy	1.083	*P*4/*mmm*	11.6419 (6)		7.5033 (3)	1017.0 (1)	3a_p_ × 3a_p_ × 2a_p_	5.35 (1)	0.33 (3)
Y	1.075	*P*4/*mmm*	11.6263 (2)		7.5030 (1)	1014.18 (5)	3a_p_ × 3a_p_ × 2a_p_	5.34 (1)	0.24 (2)

a Lattice parameters are obtained from Rietveld analysis of laboratory XRD data. The oxygen content after synthesis was determined using iodometric titration. Changes in oxygen content (Δ oxygen content) are from TGA measurements between RT and 1000 °C under flowing N_2_. Ionic radii of the Ln^3+^ cation are from ref (62)

**Figure 3:**
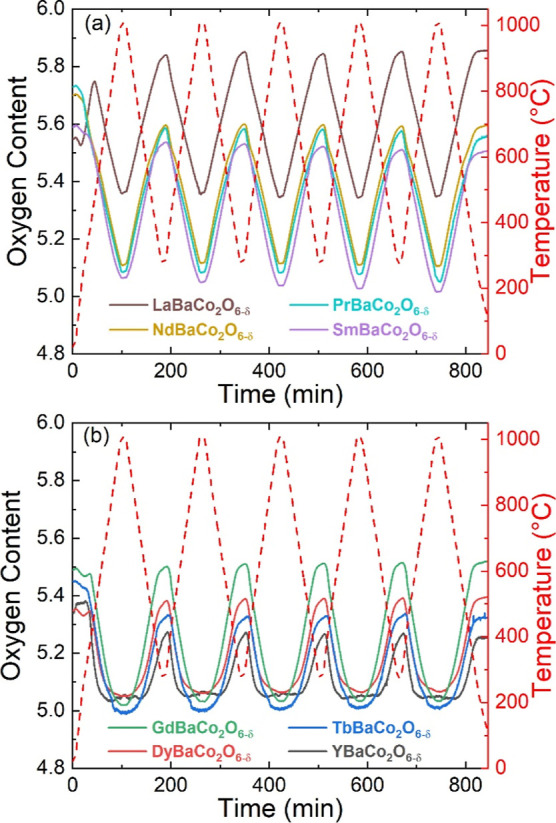
Overview of the oxygen cycling performance of the layered LnBaCo_2_O_6-δ_ perovskites for (a) Ln = La, Pr, Nd, and Sm and (b) for Ln = Gd, Tb, Dy, and Y. Data are shown as a function of time over five heat–cool cycles (RT-1000 °C, then cycling between 300 and 1000 °C) obtained by TGA in flowing N_2_. Starting values for δ are determined via iodometric titration.

Figure 3 displays the change in oxygen content for the LnBaCo_2_O_6-δ_ compositions over five heat–cool cycles (RT-1000 °C, then 300–1000 °C) in flowing N_2_ from TGA measurements. All samples follow the temperature profile in producing oxygen vacancies during heating and gaining oxygen during cooling. Rietveld analysis revealed nearly unchanged lattice parameters (volume change <0.5%) and no detectable decomposition products after cycling (Table S1). Compositions with Ln between La and Gd show similar oxygen releases and uptakes of ∼0.5 mol of O (∼3.2 wt %) with no decrease in performance over the five heat–cool cycles. However, the upper and lower δ limits between which cycling occurs significantly change, as illustrated in Figure 2d. This is consistent with the increased levels of oxygen vacancies in the as-formed materials, with cycling occurring in a comparable bandwidth until δ = 1 is reached. This occurs for the Dy and Y materials, which have much-reduced oxygen cycling capacities. For example, YBaCo_2_O_6-δ_ with δ = 0.66 after synthesis has a capacity of only ∼0.24 mol O (1.6 wt %), cycling between 0.75 < δ < 1. In fact, from the NPD analysis below, this composition reaches δ = 1 at 500 °C under flowing N_2_ and cannot release any more oxygen, instead undergoing decomposition.

### Oxygen Migration Pathways for LnBaCo_2_O_6-δ_

**Figure 4:**
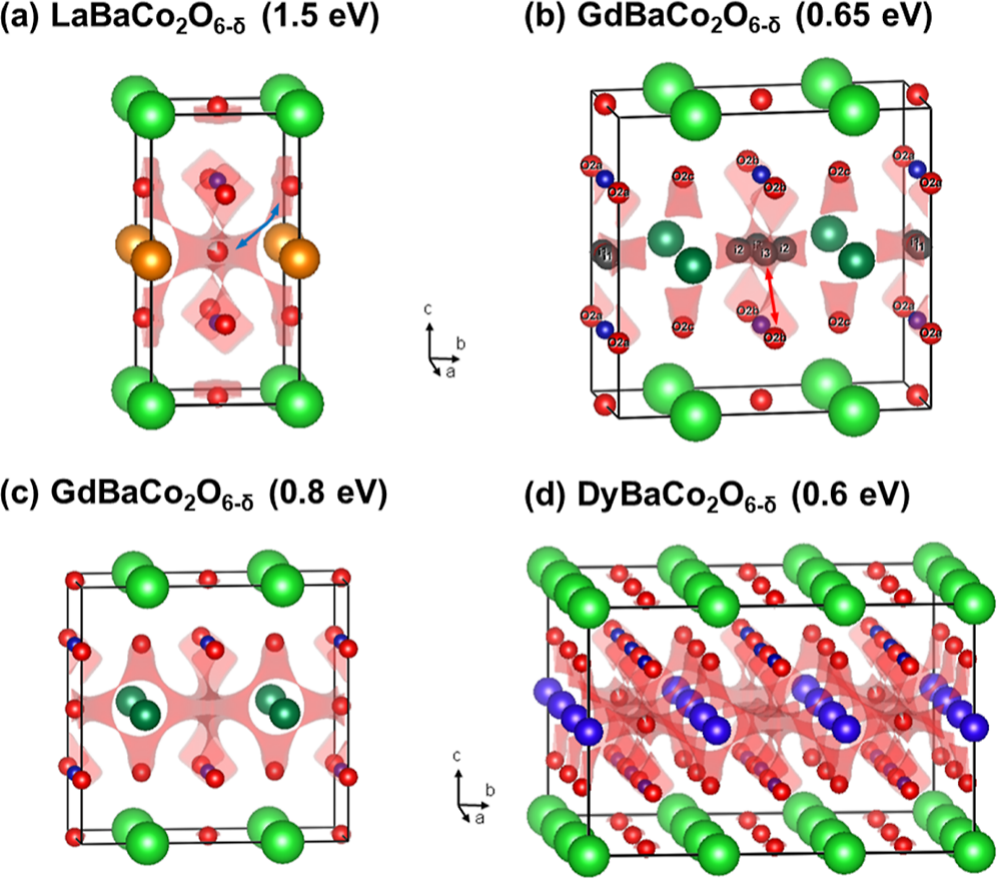
Bond valence site energy (BVSE) isosurface maps for (a) 2D oxygen migration in 1 × 1 × 2 LaBaCo_2_O_6-δ_, (b) 1D migration in 1 × 2 × 2 GdBaCo_2_O_6-δ_, (c) 2D migration in 1 × 2 × 2 GdBaCo_2_O_6-δ_, and (d) 2D migration in 3 × 3 × 2 DyBaCo_2_O6_–δ_. Ba, Co, and O atoms are colored light green, blue, and red; La, Gd, and Dy are gold, dark green, and purple. Local minima in (b) are i1 (0.41, 0, 0.5) is near O3a (0.5, 0, 0.5); i2 and i3 are located near vacant O3b (0.5, 0.5, 0.5) with coordinates: i2 (0.5, 0.43, 0.5) and i3 (0.35, 0.5, 0.5). Local minima are not shown in (c) for clarity. Migration in the 3 × 3 × 2 superstructure also involves local minima near vacant oxygen sites and is discussed in detail for YBaCo_2_O6_–δ_. Blue and red arrows in (a) and (b) indicate O2–O3 (2D) and O2b-i3 (1D) migration paths.

**Figure 5:**
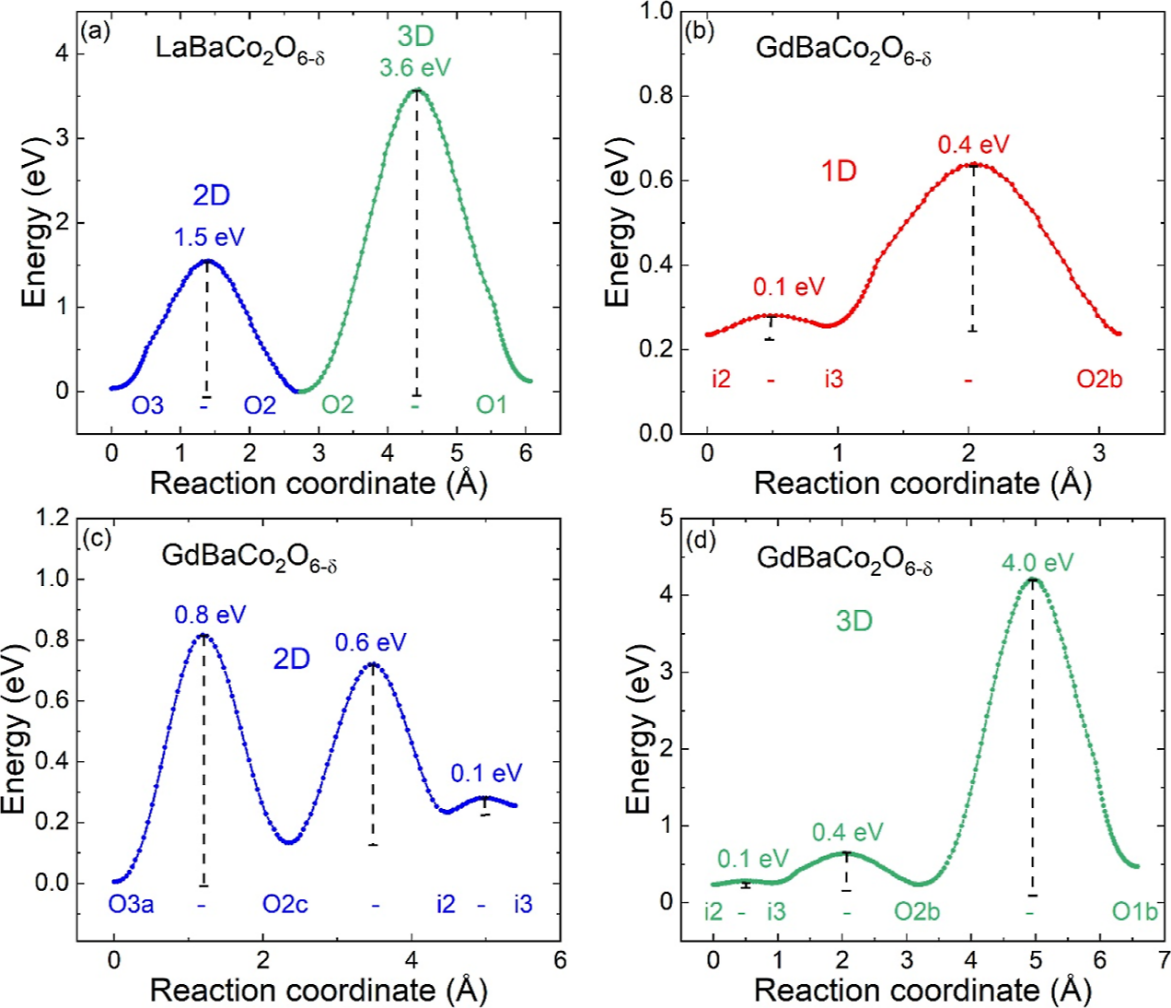
Migration barriers (*E*_*b*_) for (a) 2D and 3D oxygen migration in tetragonal 1 × 1 × 2 LaBaCo_2_O_6-δ_. (b,c,d) for 1D, 2D, and 3D migration in orthorhombic 1 × 2 × 2 GdBaCo_2_O_6-δ_ involving oxygen sites and local minima. LaBaCo_2_O_6-δ_ and GdBaCo_2_O_6-δ_ are representative of the 1 × 1 × 2 and 1 × 2 × 2 LnBaCo_2_O_6-δ_ systems, respectively. The coordinates for the local minima for GdBaCo_2_O_6-δ_ are given in the caption of Figure 4

**Figure 6:**
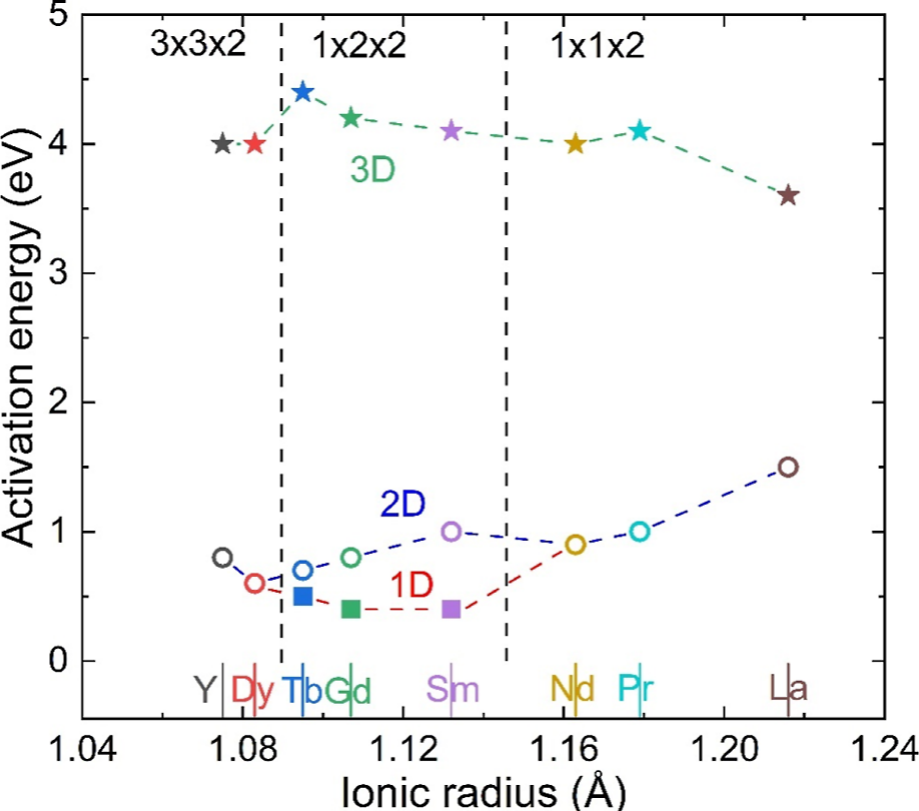
Overview of BVSE migration barrier energies (*E*_*b*_) for 1D, 2D, and 3D oxide ion transport in the LnBaCo_2_O_6-δ_ series plotted against ionic radius of the lanthanides.

BVSE isosurfaces and migration barriers are shown in Figures 4 and 5 for each structure type. The basic tetragonal structure has low energy migration pathways in the *ab* plane, with oxygen migration using both the O3 site in the Ln-O layer and the O2 site in the octahedral basal plane. This leads to a zigzag path involving···O2–O3–O2–O3···sites, in agreement with the literature. This lowest energy pathway has a single migration barrier (*E*_*b*_), decreasing from 1.5 eV (La) to 1 eV (Pr) and 0.9 eV (Nd) (Figure 6 and Table S2). The orthorhombic 1 × 2 × 2 distortion (Ln = Sm, Gd, or Tb) allows ionic migration along the *a*- and *b*-directions to be different, leading to two 1D paths. One of these has a substantial lower *E*_*b*_ = 0.6–0.4 eV (along the *a* direction), while the other (along the doubled and elongated *b* axis) is increased (Figure 6). However, even the “high” energy 1D barriers *E*_*b*_ = 1.0–0.7 eV are still reduced in comparison with the 2D barriers for Ln = La, Pr, and Nd, hence affording 2D conduction at lower overall migration barrier energies. The BVSE calculations reveal the presence of several local minima (*i*) near the vacancy ordered (largely empty) O3b site in the Ln-O plane. These replace the O3b site itself as the lowest energy position for oxide ions in the 1D and 2D migration paths, but they still have increased energy compared to that of the filled O3a site. Overall, we can conclude that 1D migration can occur at strongly reduced migration barriers (∼0.5 eV) for Ln = Sm–Tb, and that even 2D migration, which requires the higher energy 1D path to become active, has comparable or lower *E*_*b*_ than the earlier Ln = La–Nd.

The occurrence of distinct 1D migration pathways in the 1 × 2 × 2 structure has also been observed using DFT. For Ln = Pr, the reported results agree with our BVSE calculations with a reduced *E*_*b*_ = 0.5 eV along the *a* direction and an increased *E*_*b*_ = 0.9 eV along the elongated *b* axis.^28^ By contrast, for Ln = Gd, the reverse has been reported, with a high *E*_*b*_ = 1.8 eV along the *a* direction and a reduced *E*_*b*_ = 1.0 eV along the doubled *b* direction.^27^ From our BVSE calculations, the lattice distortion and position of the Ln ion are the main drivers for the emergence of two different 1D paths. In particular, if the lattice expands and the Ln ions move outward along the *b* direction, this will reduce *E*_*b*_ in the *a* direction, by reducing steric hindrance from the Ln cation.^23^ Hence, the specifics of the structural relaxation in DFT becomes important, but this information is not typically provided.^27,28^ DFT was used by another group to study the basic 1 × 1 × 2 structure (Ln = Pr), yielding *E*_*b*_ = 0.9 eV for the O2–O3 path,^29^ in good agreement with our BVSE calculations. A final point of interest is that DFT and MD simulations can find comparable migration barriers for O2–O2 hopping in the CoO_2_ planes,^28,32^ and sometimes even for direct O3–O3 jumps,^27^ despite the long distance (∼a_p_) between the latter. This strongly contrasts with BVSE where direct O2–O2 and O3–O3 jumps have much higher energies, and with DFT for the 1 × 1 × 2 structure, where most internal coordinates are fixed, with *E*_*b*_ = 3.9 eV (O2–O2) and 2.3 eV (O3–O3).^29^

The 3 × 3 × 2 superstructures have 2D migration pathways from BVSE, consistent with their tetragonal symmetry, with slightly increased *E*_*b*_ = 0.6–0.8 compared to the trend established by the basic tetragonal and orthorhombic superstructures (Figure 6). However, overall these are among the lowest calculated 2D *E*_*b*_ for the LnBaCo_2_O_6-δ_ materials. The precise migration pathways for the 3 × 3 × 2 superstructures will be discussed below for YBaCo_2_O_6-δ_, where accurate oxygen positions from NPD data are available.

### Variable Temperature NPD Study of YBaCo_2_O_6-δ_

**Figure 7:**
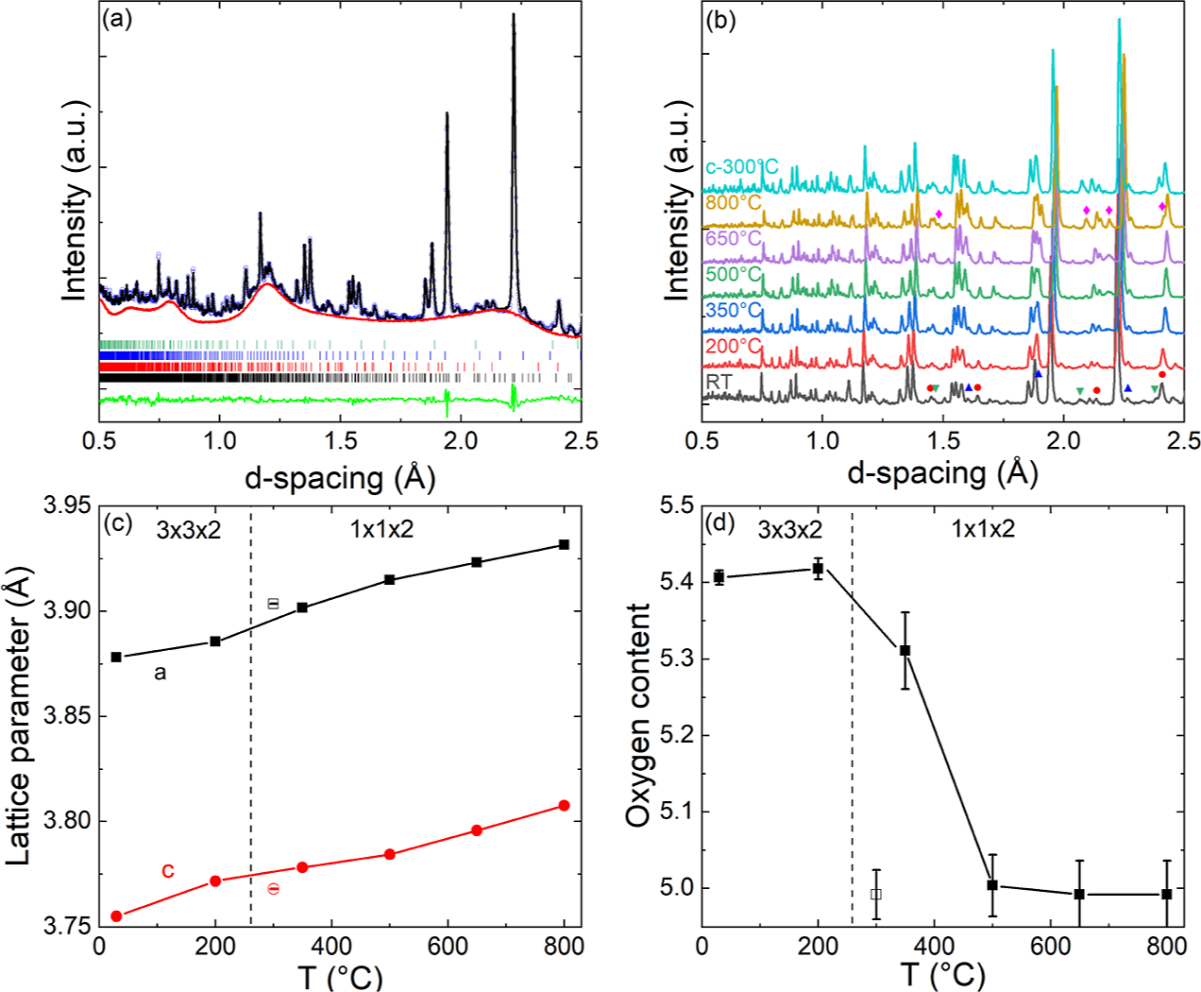
(a) Rietveld fit (black line) to GEM NPD data (open circles) collected on YBaCo_2_O_6-δ_ at RT under a N_2_ flow. The background (red line) is fixed using data from an empty sample holder, the green line is the difference curve. Reflection markers for YBaCo_2_O_6-δ_ and the impurities YBaCo_4_O_7_, Y_2_O_3_, and Co_2_O_3_ are indicated with black, red, blue, and green vertical lines. (b) Stacked NPD patterns for YBaCo_2_O_6-δ_ measured at different temperatures (RT - 800 °C) upon heating and at 300 °C after cooling under N_2_ flow. Impurity peaks of YBaCo_4_O_7_, Y_2_O_3_, Co_2_O_3_, and YBa_2_CoO_5_ are identified with symbols ●(red), ▲(blue), ▼(green), and ⧫(pink). Temperature evolution of (c) normalized lattice parameters and (d) oxygen content of YBaCo_2_O_6-δ_ from Rietveld analysis of NPD data between RT and 800 °C upon heating and at 300 °C after cooling (open symbols).

Rietveld analysis confirmed the 3 × 3 × 2 superstructure with a fitted oxygen content of δ = 0.59(1) after synthesis. Key structural information is summarized in Table 3 and the room temperature Rietveld fit is shown in Figure 7a. In this unit cell, only the O3a sites are filled, leaving 5 out of 9 of the oxygen sites in the Y–O plane empty. The vacant sites form a cross shape, consisting of two perpendicular 1D channels, as illustrated in Figure 1c. The oxygen vacancies lead to square-pyramidal CoO_5_ coordination for most of the Co ions, where the basal plane O2 anions are pulled toward the Y–O layer. Some evidence for the preferred oxygen migration paths comes from the anisotropic atomic displacement parameters that are shown in Figure S3 and summarized in Table S4. For example, U_11_(O2b) = 0.049(9) Å^2^; U_33_(O2a) = 0.023(3) Å^2^, indicating strong displacements in the *ab* plane and toward the Y–O layer. The filled O3a site shows high thermal motion in the *ab* plane [U_11_ = 0.043(7) Å^2^]. These observations are in keeping with the 2D oxygen transport mechanism involving the basal plane O2 and apical O3 oxygen sites that is well-established for the basic layered tetragonal structure.

**Table 3:** Structural Parameters for YBaCo_2_O_6-δ_ from Rietveld Fits against Neutron Powder Diffraction Data, between RT and 800 °C on Heating, and at 300 °C after Cooling^a^

	RT	200 °C	350 °C	500 °C	650 °C	800 °C	300 °C–c
supercell	3 × 3 × 2	3 × 3 × 2	1 × 1 × 2	1 × 1 × 2	1 × 1 × 2	1 × 1 × 2	1 × 1 × 2
a (Å)	11.6344 (3)	11.6566 (3)	3.9017 (1)	3.9149 (1)	3.9233 (1)	3.9316 (1)	3.9036 (1)
c (Å)	7.5099 (3)	7.5431 (3)	7.5561 (3)	7.5687 (3)	7.5912 (3)	7.6152 (3)	7.5357 (3)
V (Å^3^)	1016.53 (7)	1024.9 (1)	115.03 (1)	116.00 (3)	116.85 (1)	117.71 (1)	114.83 (1)
Occ O2	1	1	1.00 (1)	1.00 (1)	1.00 (1)	1.00 (1)	1.00 (1)
Occ O3	0.92 (2)	0.94 (3)	0.30 (2)	0	0	0	0
Ox. cont	5.41 (1)	5.42 (1)	5.31 (5)	5.00 (4)	5.00 (4)	5.00 (4)	5.00 (4)
Co^x+^	2.91 (1)	2.92 (1)	2.81 (5)	2.50 (4)	2.50 (4)	2.50 (4)	2.50 (4)
wR_P_ (%)	1.33	1.81	2.13	1.98	2.16	2.06	2.37

a Further structural information is available in Tables S2–S5. Data sets were fitted using two *P*4/*mmm* models. 3 × 3 × 2 with sites: Y1 1b (0 0 0.5); Y2 4m (0 y 0.5); Y3 4k (x x 0.5); Ba1 1a (0 0 0); Ba2 4l (0 y 0); Ba3 4j (x x 0); Co1 8r (x x z); Co2 8t (x 0.5 z); Co3 2h (0.5 0.5 z); O1a 4j (x x 0); O1b 4n (x 0.5 0); O1c 1c (0.5 0.5 0); O2a 8s (0 y z); O2b 4i (0 0.5 z); O2c 16u (x y z); O 2d 8t (x 0.5 z); O3a 4k (x x 0.5); O3b 4o (0.5 y 0.5) and O3c 1d (0.5 0.5 0.5). 1 × 1 × 2 with sites: Y 1b (0 0 0.5); Ba 1a (0 0 0); Co 2h (0.5 0.5 z); O1 1c (0.5 0.5 0); O2 4i (0.5 0 z); and O3 1d (0.5 0.5 0.5).

Stacked NPD patterns for YBaCo_2_O_6-δ_ collected between RT and 800 °C during heating and cooling under a N_2_ flow are presented in Figure 7b. The temperature evolution of the lattice parameters and oxygen content is shown in Figure 7c–d. The major fitted structural parameters are summarized in Table 3. These data reveal a phase change from the 3 × 3 × 2 superstructure to the basic 1 × 1 × 2 layered structure above 200 °C. This corresponds to an order–disorder transition of the oxygen vacancies, yielding a single O3 site that is about 25% occupied at 350 °C. At 500 °C, the O3 site is fully depopulated, corresponding to δ = 1. The oxygen content does not decrease any further up to 800 °C, consistent with YBaCo_2_O_5_ being the stable end-member composition for oxygen removal. Inspection of the lattice parameters (Figure 7c) reveals that the *c* axis expands fastest in the regions with constant oxygen content (RT - 200 and 500–800 °C), while in between, the *a* axis expands faster. This suggests a stronger impact of chemical reduction on the basal plane dimensions, whereas the *c* axis is more sensitive to thermal expansion (increasing atomic vibrations). This is also reflected in the *c/a* ratio, which increases in the regions of thermal expansion, and decreases where chemical reduction occurs (Figure S4). The sample does not regain oxygen during cooling (unlike during the TGA experiment, using the same N_2_ gas) and keeps the basic layered tetragonal structure and an unchanged (δ = 1) composition at 300 °C (Table 2). This discrepancy results from a tighter vacuum in the neutron sample environment and was also noted for our earlier investigation into the LaBaCo_2_O_6-δ_ system.^23^ The oxygen contents from TGA and NPD are in excellent agreement with the NPD data collected upon heating.

The YBaCo_2_O_6-δ_ phase proved difficult to prepare fully phase pure, in particular at the larger scale needed for NPD. After synthesis, the sample contained 14 wt % YBaCo_4_O_7_, 4 wt % Y_2_O_3_ and 2 wt % Co_2_O_3_. The amounts of YBaCo_4_O_7_ and Y_2_O_3_ are unchanged upon heating and cooling. By contrast, Co_2_O_3_ disappears at 350 °C and a YBa_2_CoO_5_ phase appears at this temperature, increasing to ∼5 wt % at 500 °C and then remaining constant (Figure S5 and Table S6). In this phase, Co is in the 3+ oxidation state and coexists with the main YBaCo_2_O_5_ (with Co^2.5+^) phase. YBa_2_CoO_5_ is not present in the diffraction data after cooling to 300 °C, showing that its formation is a reversible process, with no other new impurity peaks observed. The mechanism for the reversible formation of YBa_2_CoO_5_ is unclear, with no obvious source of Y and Ba, other than the main YBaCo_2_O_6-δ_ phase. However, decomposition of YBaCo_2_O_6-δ_ to form YBa_2_CoO_5_ would result in the formation of Co–O and Y–O phases, which were not observed.

### Bond Valence Sums of YBaCo_2_O_6-δ_

Bond valence sums (BVSs)^39^ provide a useful validation of the Co-oxidation states as oxygen is removed under N_2_ flow. In the 3 × 3 × 2 superstructure, the calculations (Table S7) show that the oxygen vacancy ordering is coupled to a charge ordering of the Co cations. The Co1 ions with octahedral coordination have a high oxidation state +3.6, while Co2 and Co3 with square-pyramidal coordination have oxidations state of +2.6 and +2.8. The average BVS oxidation state of +3.0 is in good agreement with the value of +2.9 calculated from the nominal stoichiometry δ = 0.59(1). At 500 °C (using a correction for thermal expansion, see the Experimental Section),^44^ the Co-oxidation states decreases to +2.6, in agreement with the expected Co^2.5+^ oxidation state for δ = 1. These calculations also reveal that Y is under bonded, consistent with the large concentration of oxygen vacancies in the Y–O and adjacent Co–O2 layers. Ba appears to be over bonded, which is caused by the presence of a few short Ba–O contacts, likely occurring to compensate for the displacement of the O2 anions toward the Y–O layer.

### Oxygen Migration Pathways for the 3 × 3 × 2 and Regular Structure of YBaCo_2_O_6-δ_

**Figure 8:**
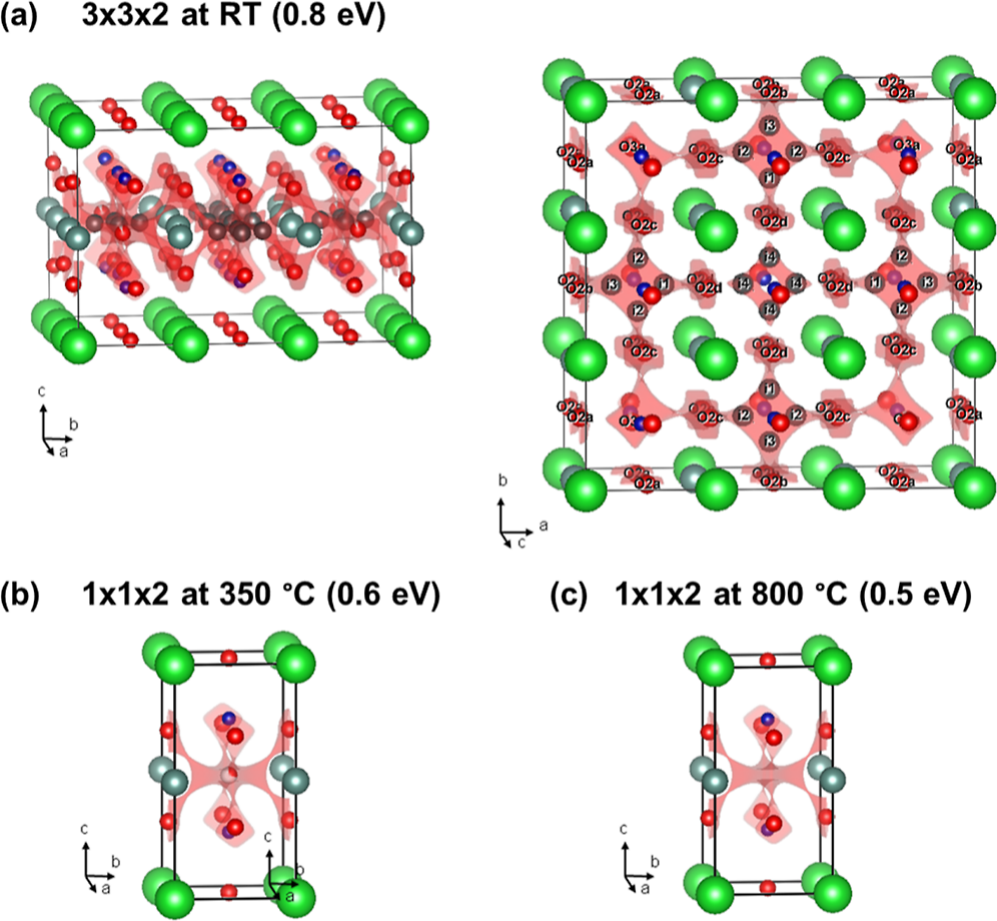
BVSE isosurface maps for 2D oxygen migration in YBaCo_2_O_6-δ_ shown for (a) the 3 × 3 × 2 superstructure at RT, viewed along the *a*- and *c*-axis, (b) the 1 × 1 × 2 structure at 350 °C and (c) at the 800 °C with depleted O3 site. Y, Ba, Co, and O are colored gray, orange, blue, and red, respectively. Coordinates of the local minima in (a) are i1 (1/2, 0.23, 1/2); i2 (0.16, 0.43, 1/2); i3 (1/2, 0.09, 1/2); and i4 (0.43, 1/2, 1/2). Minima i1–i3 surround the vacant O3b site (1/2, ∼ 0.17, 1/2), while i4 is next to the vacant O3c site in the body center (1/2, 1/2, 1/2).

**Figure 9:**
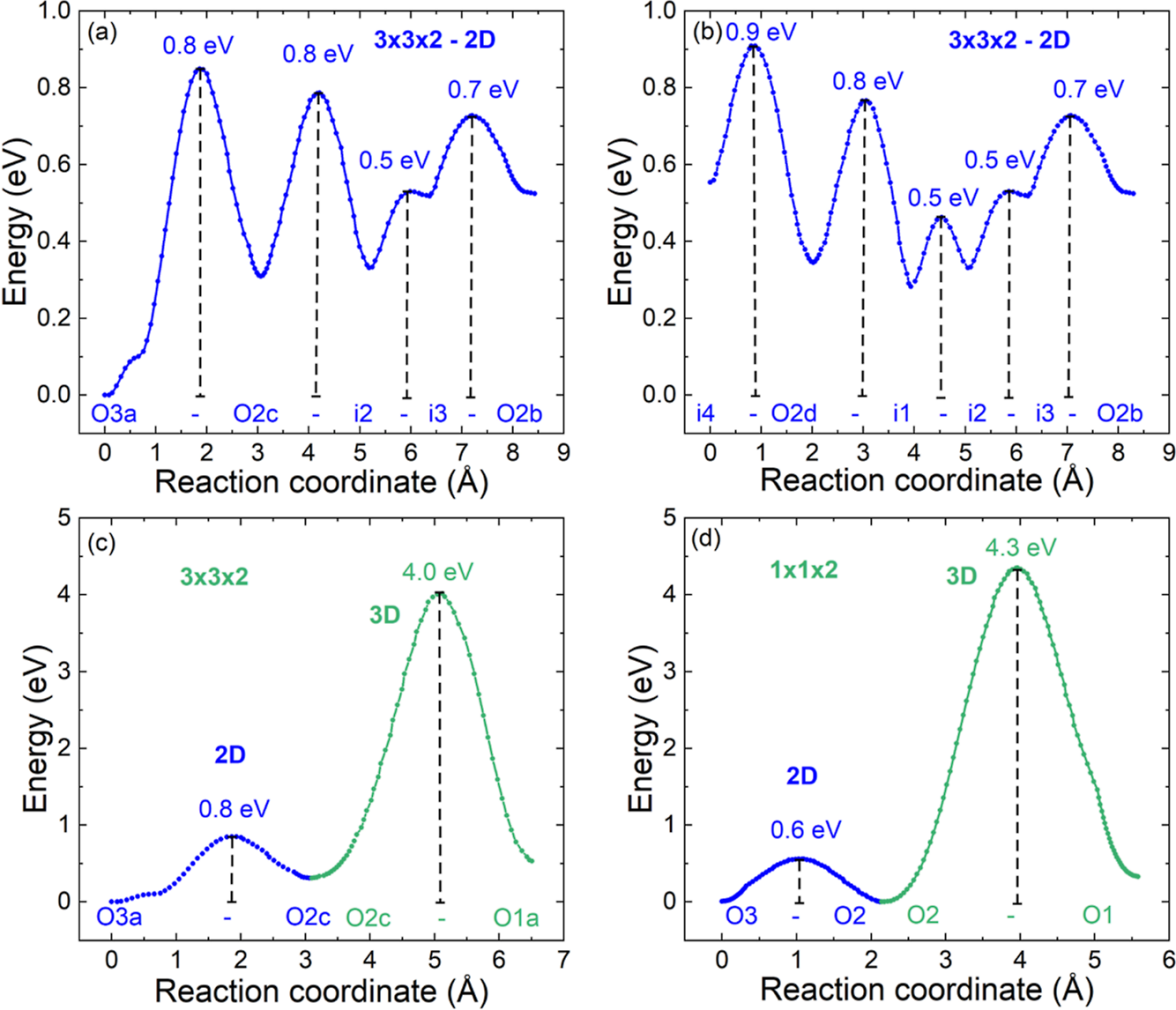
BVSE migration barriers (*E*_*b*_) for oxide ion transport in YBaCo_2_O_6-δ_. Panels (a–c) show low energy migration pathways for the 3 × 3 × 2 superstructure at RT. Panel (d) shows data for the basic 1 × 1 × 2 structure at 350 °C. Panel (a) shows the lowest energy migration (*E*_*b*_ = 0.8 eV) path connecting the O3a-O2c-i2-i3- O2b sites. Panel (b) shows the slightly higher energy (*E*_*b*_ = 0.9 eV) path connecting i4-O 2d-i1-i2-i3-O2b sites. The latter path involves the i4 mimima that surround the body centered vacant oxygen site (O3c) in the unit cell. Coordinates for the local minima are given in the caption of Figure 8

Constant energy isosurfaces and low energy oxide ion migration pathways are shown in Figures 8 and 9. These calculations are based on the structures fitted against NPD data, including the accurate oxygen positions. From 350 °C, YBaCo_2_O_6-δ_ has the basic layered 1 × 1 × 2 structure with ionic migration involving hopping between O2 and O3 sites. This transport is isotropic in the *ab* plane with *E*_*b*_ = 0.6 eV at 350 °C, decreasing to 0.5 eV at 800 °C, consistent with the expected impact of thermal expansion. 3D transport involves crossing the Ba–O plane and carries a large energy penalty, *E*_*b*_ = 4.3 eV, due to the large size of the Ba^2+^ cations. The low *E*_*b*_ for 2D migration is consistent with the common observation of low activation energies in EIS for Ln = Y (Table 1).

The 3 × 3 × 2 superstructure has two perpendicular channels of ordered vacancies, which may a priori be expected to provide facile pathways for oxygen ion migration. However, ionic motion proceeds in the same zigzag manner between the O2–O3 sites, with no direct movement within the vacancy channels. The ionic transport is similar to the basic 1 × 1 × 2 structure but now involves a much larger number of distinct O2 and O3 positions. The lowest energy (2D) pathway (*E*_*b*_ = 0.8 eV) involves O2b, O2c, and O3a sites, as well as local minima i2 and i3 adjacent to the vacant O3b site (Figures 8 and 9). A slightly higher energy pathway (*E*_*b*_ = 0.9 eV) includes the i4 minima near the O3c site at the body center of the unit cell and the adjacent O 2d sites. The O2a site is not involved in oxygen migration at low activation barriers. The local minima have energies of 0.35–0.7 eV (compared to filled O3a), reflecting the energy penalty of placing oxygen ions on vacant oxygen sites. This is particularly pronounced for the O3c site, which is not part of the ionic migration pathway and is circumvented by using the adjacent i4 minima. This shows that there is a “blockage” at the body center of the unit cell, where the two 1D vacancy channels intersect. The intuitively appealing picture of two empty vacancy channels supporting ionic transport therefore does not hold. This is because the Y^3+^ cations remain in place and they dictate the magnitude of *E*_*b*_, forcing the zigzag motion involving the O2 and the O3 sites rather than direct hopping between the O3 sites. Significantly, the higher temperature vacancy disordered structure affords substantially lower *E*_*b*_ < 0.6 eV for 2D migration. This reduction is much larger than expected from thermal expansion. Hence, the 3 × 3 × 2 vacancy ordering is not favorable for ionic transport. A trial BVSE calculation based on the 1 × 1 × 2 structure but using the RT lattice parameters (*a*/3, *c*) and 350 °C atomic coordinates, yields *E*_*b*_ = 0.63 eV for 2D migration (and 4.4 eV for 3D). This demonstrates that the increased *E*_*b*_ is not caused by changed cell metrics but must result from changes in the internal coordinates. Inspection of the 3 × 3 × 2 and 1 × 1 × 2 structures shows that O2 ions are substantially displaced toward the Y–O plane (Figure 8). This causes increased steric hindrance due to a closer proximity to the Y ions, consistent with the larger calculated *E*_*b*_ for the 3 × 3 × 2 structure.

To summarize, BVSE confirms the presence of the basic zigzag. ···O2–O3–O2···migration pathway in YBaCo_2_O_6-δ_. Initially, this involves the O3a-O2c-i2-i3-O2b sites with *E*_*b*_ = 0.8 eV. At slightly higher energies (*E*_*b*_ = 0.9 eV), the O 2d and i4 sites become accessible, activating the i4-O 2d-i1-i2-i3-O2b channel, which partially involves the same sites as the lower energy path. The lowest energy 3D transport pathway across the Ba–O layer (from O3a-O2c-O1a) has *E*_*b*_ = 4 eV and is comparable to the basic layered structure (Figure 9). Increasing the temperature causes the oxygen vacancies to disorder and substantially reduces the 2D migration barrier to *E*_*b*_ = 0.6 eV at 350 °C.

## Discussion

The main outcome of this study is a better understanding of the evolution of ionic migration pathways of the LnBaCo_2_O_6-δ_ perovskites. The most important factor is the size of the Ln cation with oxygen vacancy ordering, offering some interesting prospects near room temperature. For Ln = La and Y, the vacancies are known to disorder above 400 and 200 °C, and any vacancy ordered structure for intermediate Ln is likely to have a similar temperature stability. Hence, the superstructures are relevant only for low temperature applications. Above 300–400 °C, the structure is the basic layered 1 × 1 × 2 structure. The BVSE results show a global reduction in *E*_*b*_ for 2D transport with decreasing Ln size. At room temperature, this trend is somewhat obscured by the occurrence of the 1 × 2 × 2 and 3 × 3 × 2 superstructures with their more complicated migration pathways. However, the minimum barrier for 2D transport can clearly be seen to decrease as the Ln radius decreases (Figure 6). This happens because the smaller Ln cause less steric hindrance for the O2–O3 motion that underpins oxygen migration. In this regard, the presence of Ba in separate layers is vital as this serves to keep the basal plane dimensions expanded as the size of the Ln decreases. Trial calculations using the 1 × 1 × 2 cell of Ln = Pr but with Y included in the structure yield a halved *E*_*b*_ = 0.5 eV for the O2–O3 jump, thus confirming the key importance of Ln size. The 3D barrier *E*_*b*_ = 4.1 eV remains unchanged. Trial BVSE calculations for Ln = Pr with a 5% expanded *ab* plane also yield *E*_*b*_ = 0.5 eV for the jump between O2 and O3 with the barrier for 3D transport reduced to *E*_*b*_ = 2.4 eV, as the Ba–O layer expands and opens-up. This confirms that expanding the *ab* plane dimensions is key for reducing migration barriers. By contrast, expanding the lattice in the *c* direction by 5% only results in a ∼ 10% reduction to *E*_*b*_ = 0.9 eV for the O2–O3 jump and to *E*_*b*_ = 3.4 eV for 3D migration. Overall, it can be concluded that the key design criterion for the 1 × 1 × 2 structure is to have the largest possible mismatch between the *ab* plane dimensions and the Ln cation size. The *ab* plane is largely fixed by the large Ba cation and shrinks only moderately with decreasing Ln size (Figure 2). Hence, the YBaCo_2_O_6-δ_ composition should have the highest ionic conduction. The reason that this is not observed is that the 3 × 3 × 2 vacancy ordering leads to increased *E*_*b*_. In addition, the Ln = Y composition is at the stability limit of the structure, and on these grounds, larger Ln might be preferred. Partial replacement of Ba by Sr is known to increase structural stability for smaller Ln.^45^ However, the resulting decrease in the *ab* plane dimension will negatively affect *E*_*b*_ because of increasing steric hindrance due to the closer proximity of the Ln cations. Interestingly, Sr-substitution may be beneficial for 3D migration across the Ba–O plane due to its smaller size. A trial calculation, again using the 1 × 1 × 2 cell of Ln = Pr but replacing Ba by Sr leads to a calculated halved *E*_*b*_ = 2 eV for 3D transport. This reduced value only holds for the Ba compound, whereas in reality, substitution with Sr would lead to a shrinkage of the *ab* plane, offsetting the impact of the smaller size of Sr.

The vacancy ordered superstructures have a contrasting impact on ionic migration barriers. The 1 × 2 × 2 materials (Ln = Sm–Tb) have 1D channels with low overall *E*_*b*_ ∼ 0.5 eV. These occur due to reduced steric hindrance from the Ln cation, which essentially move outward along the doubled and elongated *b*-axis. Depending on the connectivity of these 1D paths in bulk samples, this can potentially afford high ionic conductivities. It is worth noting that bulk polycrystalline samples have high ionic conduction, despite the 2D nature of ionic migration. This shows that transport across the boundaries between polycrystalline grains is not an insurmountable problem. The 1D vacancy ordered structures therefore offer the potential for improved ionic transport at low temperatures, over what is possible using the basic tetragonal 1 × 1 × 2 structure. By contrast, the 3 × 3 × 2 superstructure does not support new low energy migration pathways, with low *E*_*b*_ ∼ 0.6 eV only found for Ln = Y upon transforming to the 1 × 1 × 2 structure. The probable cause of this effect is the encroachment of the O2 layers toward the central Y–O plane that is a feature of the 3 × 3 × 2 superstructure with its many depleted O3 sites. This increases steric hindrance, due to the closer proximity of Y to the O2 sites has a net negative effect on *E*_*b*_.

The cycling study demonstrates that as the Ln become smaller, the materials have a narrower oxygen stability window, centered on an increasing δ value with a hard limit of δ = 1. The implication is that for smaller Ln, the Co ions remain in a higher oxidation state as the materials are cycled. This provides an explanation for the reduced thermal expansion that is often seen as one of the major advantages of smaller Ln. However, our work demonstrates that the smaller Ln also has the inherently lowest migration barriers for oxide ion transport.

To conclude, this work provides new insights and guidance for further materials design work. In particular, the impact of the low energy 1D channels in the orthorhombic 1 × 2 × 2 structures should be explored. For the basic tetragonal materials, finding the best trade-off between the expansive effect of the Ba–O layer and having a small Ln cation, is the key challenge, with limited impact from expanding the lattice in the *c* direction. Mixing larger and small Ln (e.g., La/Y) is therefore not expected to be beneficial. Replacing Co with larger ions (e.g., Fe) may be a fruitful route toward improvement. The general approach outlined in this study can readily be expanded to include related LnBaM_2_O_6-δ_ (M = Mn and Fe) and mixed cation systems. From the materials studied, here Ln = Sm and Gd outperform the other compounds with low activation barriers for 1D and 2D oxygen migration, good stability, and oxygen cycling capacity.

## Experimental Section

### Synthesis

5-g polycrystalline LnBaCo_2_O_6-δ_ (Ln = La, Pr, Nd, Sm, Gd, Tb, Dy, and Y) samples were prepared by the solid-state reaction. La_2_O_3_ (Sigma-Aldrich, 99.999%), Pr_6_O_11_ (Alfa Aesar, 99.99%), Nd_2_O_3_ (Alfa Aesar, 99.99%), Sm_2_O_3_ (Alfa Aesar, 99.99%), Gd_2_O_3_ (Alfa Aesar, 99.99%), Tb_2_O_3_ (Sigma-Aldrich, 99.99%), Dy_2_O_3_ (Alfa Aesar, 99.99%), and Y_2_O_3_ (Sigma-Aldrich, 99.999%) were used as the lanthanide precursor. Stoichiometric amounts of lanthanide oxides, Co_3_O_4_ (Alfa Aesar, 99.9985%) and BaCO_3_ (Alfa Aesar, 99.997%), were mixed using a mortar and pestle and annealed in a muffle furnace for 12 h at 1000 °C. Pellets of the annealed mixture were sintered under air at 1100 °C for 12 h with heating and cooling rates of 10 °C min^–1^.

### Characterization

Initial phase analysis was undertaken by using XRD using a Bruker D8 Advance diffractometer with monochromated Cu–K_α1_ radiation. High quality data sets were collected over 7 h. Iodometric titration was used to evaluate the Co-oxidation state and determine the O content after synthesis. Four M HCl solution was saturated by bubbling Ar through the solution for a minimum of 30 min. 1 g of KI and ∼20 mg of perovskite oxide was dissolved in the HCl solution and titrated against 0.01 M Na_2_S_2_O_3_ solution under an argon atmosphere. The reported error (given in Table 2) is the standard deviation between three concordant measurements. TGA data were collected under flowing N_2_ (BOC Oxygen Free Nitrogen, p_O2_ ≈ 10^–5^ atm, 100 cm^3^ min^–1^) by using a Linseis STA PT 1600 instrument. Measurements were done on ∼200 mg of sample contained in an alumina crucible.

### Neutron Powder Diffraction

Time-of-flight NPD data were collected on around 5 g of powdered YBaCo_2_O_6-δ_ sample using the GEM diffractometer at the ISIS Neutron and Muon Source, Rutherford Appleton Laboratory, UK. The sample was loaded into a double walled quartz gas-flow holder and heated between 25 and 800 °C under N_2_ flow, using the same N_2_ gas and flow as used in the TGA experiment. Data were collected for ∼350 μAh of proton beam current, corresponding to ∼2 h exposure at each temperature. Background measurements on an empty quartz holder were carried out at 25 °C, 400 and 700 °C. These data sets were used to fix the background in the Rietveld analysis of the data sets collected on YBaCo_2_O_6-δ_. All Rietveld analysis was carried out using the GSAS II software.^46,47^ A small linear absorption correction (μR of 0.2) was applied. There was no evidence of metal deficiency on the Co sites in the structure from the Rietveld fits. The crystal structures were visualized using the VESTA software.^48^

### Bond Valence Sum and BVSE Calculations

Bond-valence sum (BVS) calculations were used to determine the oxidation states of metal cations and oxygen anions.^39^ The following BVS parameters for Co were used: R_0_ = 1.74 and B = 0.37,^23^ which are typical of high-spin Co^3+^, as expected above RT. Bond-valence parameter of other cations were taken from the database by Gagne et al.^38^ BVSE calculations were undertaken using the SoftBV software,^36^ with the BVS parameter file updated to reflect the values determined for Co. For the LnBaCo_2_O_6-δ_ series, room temperature unit cell data obtained from fitting against laboratory X-ray diffraction data were used. In these fits, the lattice parameters and positions of the metals were allowed to vary freely, but the oxygen coordinates were kept at their pseudocubic positions. This decision was taken because the 1 × 2 × 2 and 3 × 3 × 2 structures contain a larger number of oxygen coordinates (4 and 13, respectively), making reliable refinement against laboratory data challenging. Where higher quality neutron data sets are available (Ln = La,^23^ Pr, Nb,^33,34^ and Y from this work), the results are within 0.2 eV of the values obtained with fully refined structures. For the high temperature NPD data sets, the BVS parameters were corrected for thermal expansion.^44^ A linear thermal expansion coefficient, α = 1.64 × 10^–5^ K^–1^, was determined for YBaCo_2_O_6-δ_, as outlined in the Supporting Information (Table S8 and Figure S6). BVSE maps are calculated with a resolution of 0.01 Å and plotted as constant energy isosurfaces or as plots of energy versus reaction coordinate for low-energy oxygen migration paths.

## Data Availability

Data underpinning this publication can be accessed at https://doi.org/10.17630/9aef7c64-45fa-4ecf-b9b9-08e100a47f10.

## Supporting Information Available

Supporting Information including detailed crystallographic data is available online Tables with lattice parameters, cell volumes, BVSE migration barriers, atomic coordinates, anisotropic thermal displacement parameters, impurity weight fractions, bond valence sums, and thermal expansion fit parameters; Figures with Rietveld fits to X-ray powder diffraction data, three-dimensional ellipsoidal representations of thermal displacement parameters, normalized c/a ratio, temperature evolution of impurity phase weight fractions, and thermal expansion fits (PDF)
