# Supplementary material for: Oxygen Migration Pathways in Layered LnBaCo2O6-δ (Ln = La – Y) Perovskites
Source: JACS Au. 2024 Apr 2;4(4):1538–49. doi: 10.1021/jacsau.4c00049 (PMC11040552; doi:10.1021/jacsau.4c00049)
Supplement: Supplementary file 1 — au4c00049_si_001.pdf [file au4c00049_si_001.pdf]

Supplementary information for

Oxygen migration pathways in layered $LnBaCo_2O_{6-\delta}$ (Ln = La – Y) perovskites

Fabian Hesse,[a] Ivan da Silva,[b] Jan-Willem G. Bos*[c]

[a] *Institute of Chemical Sciences, School of Engineering and Physical Sciences, Heriot-Watt University, Edinburgh, EH14 4AS, UK.*

[b] *ISIS Facility, Rutherford Appleton Laboratory, Harwell Oxford, Didcot OX11 0QX, UK.*

[c] *EaStCHEM School of Chemistry, University of St Andrews, North Haugh, St Andrews, KY16 9ST, UK.*

*Email: j.w.g.bos@st-andrews.ac.uk

Table S1. Lattice parameters and cell volumes of the $LnBaCo_2O_{6-\delta}$ samples after five heat-cool cycles (RT-1000 °C) in the TGA under flowing N_2. The volume change is compared to the same samples before TGA.

Perovskite oxides	Lattice parameters (Å)			Volume	Volume
	A	b	c	(Å^3)	change (%)
$LaBaCo_2O_{6-\delta}$	3.9091(2)		7.7663(4)	118.68(2)	-0.04
$PrBaCo_2O_{6-\delta}$	3.9061(2)		7.6056(4)	116.05(2)	0.43
$NdBaCo_2O_{6-\delta}$	3.9061(2)		7.6050(3)	116.04(2)	0.02
$SmBaCo_2O_{6-\delta}$	3.8909(2)	7.8431(5)	7.5605(5)	230.72(4)	0.04
$GdBaCo_2O_{6-\delta}$	3.8793(2)	7.8321(4)	7.5346(3)	228.92(3)	0.06
$TbBaCo_2O_{6-\delta}$	3.8913(6)	7.7904(9)	7.5285(9)	228.22(9)	-0.32
$DyBaCo_2O_{6-\delta}$	11.6486(3)		7.5091(2)	1018.91(7)	-0.19
$YBaCo_2O_{6-\delta}$	11.6207(7)		7.4961(4)	1012.3(2)	0.19

Table S2: BVSE migration barriers (E_b) for 1D, 2D and 3D oxygen transport in the layered $LnBaCo_2O_{6-\delta}$ perovskites.

Ln	1D (eV)	2D (eV)	3D (eV)
La		1.5	3.6
Pr		1.0	4.1
Nd		0.9	4.0
Sm	0.4	1.0	4.1
Gd	0.4	0.8	4.2
Tb	0.5	0.7	4.4
Dy		0.6	4.0
Y		0.8	4.0

Table S3. Atomic coordinates for $YBaCo_2O_{6-\delta}$ at RT and 200 °C upon heating.

	RT	200 °C
Y2 (y)	0.3072(6)	0.3073(8)
Y3 (x/y)	0.3296(6)	0.3297(8)
Ba2 (y)	0.336(1)	0.336(2)
Ba3 (x/y)	0.3354(9)	0.335(1)
Co1 (x/y)	0.1567(9)	0.157(1)
Co1 (z)	0.248(2)	0.248(2)
Co2 (x)	0.165(1)	0.165(2)
Co2 (z)	0.264(2)	0.264(2)
Co3 (z)	0.251(4)	0.251(7)
O1a (x/y)	0.170(1)	0.171(1)
O1b (x)	0.170(1)	0.170(1)
O2a (y)	0.1726(8)	0.173(1)
O2a (z)	0.2622(9)	0.262(1)
O2b (z)	0.315(2)	0.315(2)
O2c (x)	0.1670(6)	0.1669(9)
O2c (y)	0.3368(5)	0.3367(8)
O2c (z)	0.3061(7)	0.3059(9)
O2d (x)	0.3317(8)	0.332(1)
O2d (z)	0.3124(8)	0.312(1)
O3a (x/y)	0.147(1)	0.147(1)

Data were fitted using the $3a_p \times 3a_p \times 2a_p$ P4/mmm supercell with the following atomic sites: Y1 1b (0 0 0.5); Y2 4m (0 y 0.5); Y3 4k (x x 0.5); Ba1 1a (0 0 0); Ba2 4l (0 y 0); Ba3 4j (x x 0); Co1 8r (x x z); Co2 8t (x 0.5 z); Co3 2h (0.5 0.5 z); O1a 4j (x x 0); O1b 4n (x 0.5 0); O1c 1c (0.5 0.5 0); O2a 8s (0 y z); O2b 4i (0 0.5 z); O2c 16u (x y z); O2d 8t (x 0.5 z) and O3a 4k (x x 0.5).

Table S4. Anisotropic thermal displacement parameters (Å² x 100) for $YBaCo_2O_{6-\delta}$ between RT and 800 °C on heating and at 300 °C after cooling.

ADP (Å² x 100)	RT	200°C	350°C	500°C	650°C	800°C	300°C-c
U(Y)	0.5(1)	0.6(1)	1.5(1)	1.5(1)	1.7(1)	2.0(1)	1.3(1)
U(Ba)	0.4(1)	0.6(1)	1.2(1)	1.8(1)	1.9(1)	2.2(1)	1.5(1)
U(Co1)	0.3(1)	1.2(2)	2.2(1)	2.2(1)	2.7(1)	2.8(1)	1.7(1)
U_{11}(O1a)	2.6(4)	2.6(6)	1.9(1)	2.2(1)	2.7(1)	3.3(1)	1.4(1)
U_{33}(O1a)	0.2(2)	0.3(2)	1.3(2)	1.3(1)	2.1(2)	1.7(2)	1.6(2)
U_{12} (O1a)	1.8(5)	1.9(7)					
U_{11}(O1b)	1.6(4)	1.8(8)					
U_{22}(O1b)	0.6(4)	1.8(9)					
U_{33}(O1b)	0.1(1)	0.1(1)					
U_{11}(O1c)	2(1)	3(1)					
U_{33}(O1c)	0.2(1)	0.7(3)					
U_{11}(O2a)	0.6(3)	1.3(9)	2.1(1)	2.1(1)	2.4(1)	2.6(1)	1.8(1)
U_{22}(O2a)	1.4(3)	4.0(8)	1.1(1)	1.8(1)	1.8(1)	2.0(1)	0.4(1)
U_{33}(O2a)	2.3(3)	2.6(4)	3.4(1)	3.0(1)	3.4(1)	4.2(1)	2.1(1)
U_{23}(O2a)	0.2(1)	0.4(1)					
U_{11}(O2b)	4.9(9)	5(1)					
U_{22}(O2b)	0.3(5)	0.4(5)					
U_{33}(O2b)	0.3(4)	0.5(5)					
U_{11}(O2c)	1.0(2)	1.0(4)					
U_{22}(O2c)	0.5(2)	0.6(3)					
U_{33}(O2c)	1.1(3)	2.2(5)					
U_{12}(O2c)	0.2(2)	0.3(3)					
U_{13}(O2c)	0.2(2)	0.2(2)					
U_{23}(O2c)	0.2(2)	0.3(3)					
U_{11}(O2d)	1.0(3)	1.0(7)					
U_{22}(O2d)	0.6(4)	1.8(7)					
U_{33}(O2d)	1.8(4)	3.6(7)					
U_{13}(O2d)	0.4(3)	0.6(5)					
U_{11}(O3a)	4.3(7)	5(1)	17(2)				
U_{33}(O3a)	2.1(6)	4.6(9)	6(1)				
U_{12}(O3a)	3.2(8)	4.2(9)					

Table S5. Atomic coordinates for $YBaCo_2O_{6-\delta}$ between 350 and 800 °C upon heating and at 300 °C after cooling.

	350 °C	500 °C	650 °C	800 °C	300 °C-c
Co(z)	0.2580(5)	0.2573(4)	0.2582(5)	0.2582(5)	0.2595(4)
O2(z)	0.3057(1)	0.3110(1)	0.3120(1)	0.3124(1)	0.3119(1)

Data were fitted against the basic $a_p \times a_p \times 2a_p$ P4/mmm unit cell with sites: Y 1b (0 0 0.5); Ba 1a (0 0 0); Co 2h (0.5 0.5 z); O1 1c (0.5 0.5 0); O2 4i (0.5 0 z) and O3 1d (0.5 0.5 0.5).

Table S6. Impurity weight fractions for $YBaCo_2O_{6-\delta}$ between room temperature and 800 °C upon heating and at 300 °C after cooling.

Impurity (wt.%)	RT	200°C	350°C	500°C	650°C	800°C	300°C-c
Y_2O_3	4.4(1)	4.4(1)	4.3(1)	4.2(1)	4.3(1)	4.4(1)	4.3(1)
$YBaCo_4O_7$	14.0(3)	14.2(3)	15.4(3)	14.8(3)	15.2(3)	15.3(3)	16.1(3)
YBa_2CoO_5	0	0	3.6(1)	4.9(1)	5.0(1)	5.3(1)	0
Co_3O_4	2.1(1)	1.6(1)	0	0	0	0	4.3(1)

Table S7. Bond valence sums for $YBaCo_2O_{6-\delta}$ between RT and 800 °C upon heating and at 300 °C after cooling.

Site	RT	200°C	350°C	500°C	650°C	800°C	300°C-c
Y1	2.74(5)	2.74(6)	3.23(7)	3.68(8)	3.39(9)	3.4(1)	3.41(7)
Y2	2.92(5)	2.90(6)					
Y3	2.76(5)	2.75(6)					
Ba1	2.83(5)	2.83(6)	2.25(7)	2.15(8)	2.12(9)	2.1(1)	2.15(7)
Ba2	2.48(5)	2.48(6)					
Ba3	2.20(5)	2.19(6)					
Co1	3.56(5)	3.55(6)	2.95(7)	2.65(8)	2.63(9)	2.6(1)	2.63(7)
Co2	2.82(5)	2.83(6)					
Co3	2.60(5)	2.59(6)					
O1a	2.44(5)	2.43(6)	2.26(7)	2.27(8)	2.24(9)	2.2(1)	2.23(7)
O1b	2.18(5)	2.17(6)					
O1c	2.58(5)	2.56(6)					
O2a	2.61(5)	2.61(6)	2.00(7)	1.99(8)	1.99(9)	2.0(1)	2.00(7)
O2b	1.78(5)	1.78(6)					
O2c	1.90(5)	1.90(6)					
O2d	1.95(5)	1.96(6)					
O3a	2.16(5)	2.12(6)	2.21(7)				

Table S8. Linear fit parameters used to fit the volume expansion of $YBaCo_2O_{6-\delta}$ between RT – 200 °C and 500 – 800 °C. These fits were used to determine the thermal expansion coefficient (α) due to regular thermal expansion (i.e. in the absence of chemical reduction). The temperature dependence of the reduced volume is shown in Fig. S6.

	RT - 200 °C	500 – 800 °C
a_0 (Å)	3.83	3.84
b x 10^{-5} (Å/K)	6.29	6.34
α (K^{-1} x 10^{-5})	1.64	1.64

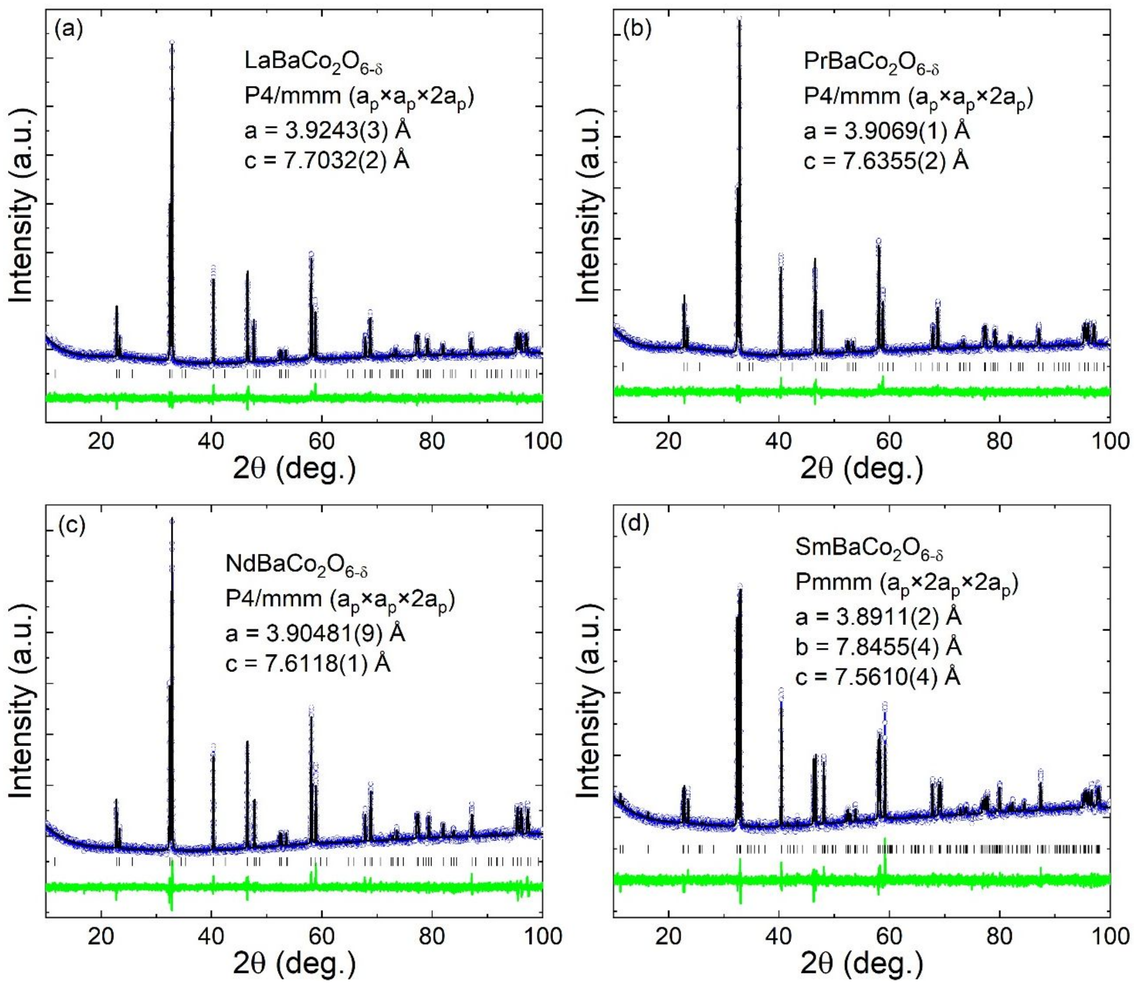

Fig. S1. Rietveld fits to X-ray powder diffraction data for $LnBaCo_2O_{6-\delta}$ (Ln = La, Pr, Nd, Sm). Data are shown open blue circles, the fit is the solid black line, and the difference curves are shown as green lines. Bragg peak positions are indicated by vertical markers. Oxygen coordinates were kept at their pseudo-cubic positions.

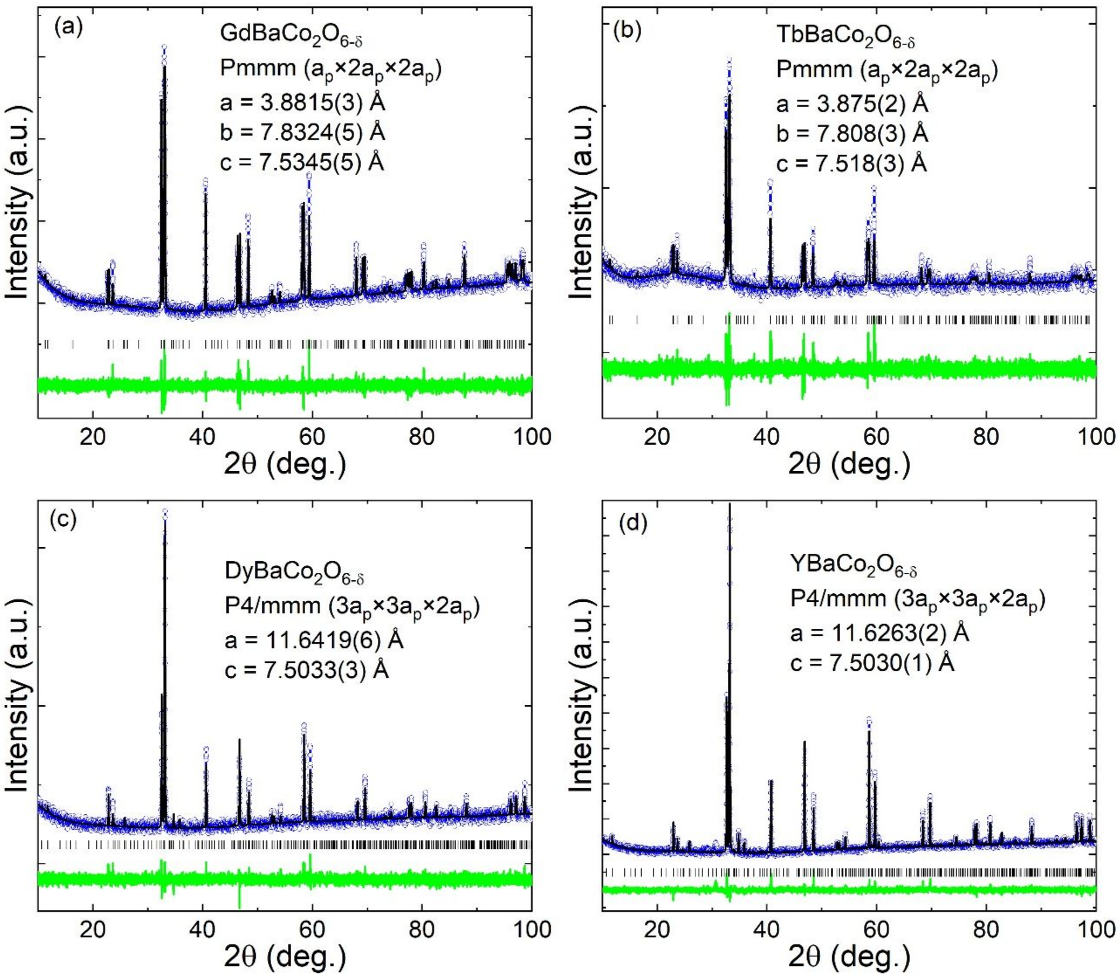

Fig. S2. Rietveld fits to X-ray powder diffraction data for $LnBaCo_2O_{6-\delta}$ (Ln = Gd, Tb, Dy, Y). Data are shown open blue circles, the fit is the solid black line, and the difference curves are shown as green lines. Bragg peak positions are indicated by vertical markers. Oxygen coordinates were kept at their pseudo-cubic positions.

(a) 3x3x2 at RT

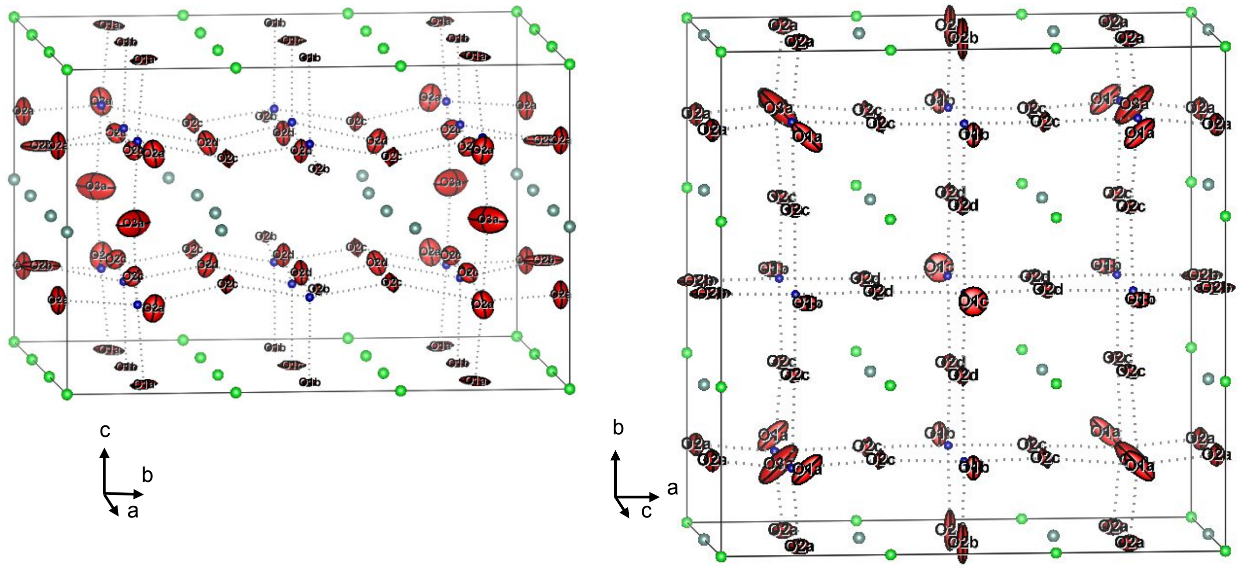

(b) 1x1x2 at 350 °C

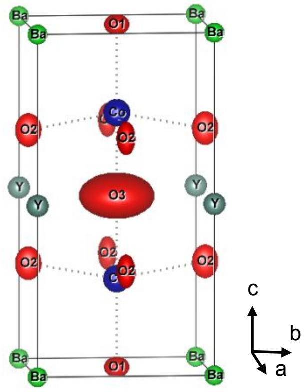

(c) 1x1x2 at 800 °C

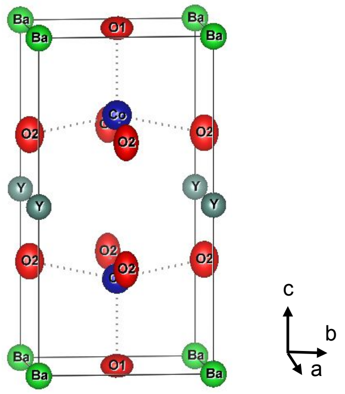

Fig. S3. Three dimensional elipsoidal representations (75% probability) of the anisotropic atomic displacement parameters found for $YBaCo_2O_{6-\delta}$ in: **(a)** the 3×3×2 superstructure at RT; **(b)** the basic 1×1×2 cell at 350 °C with partially vacant O3 site and **(c)** with empty O3 site at 800 °C. Y, Ba, Co and O atoms are coloured grey, green, blue and red, respectively.

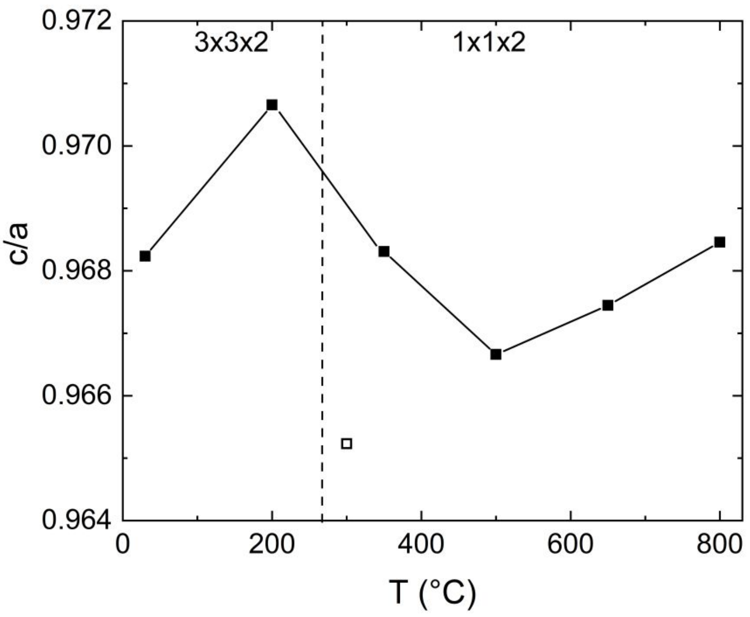

Fig. S4. Normalized c/a ratio for $YBaCo_2O_{6-\delta}$ from NPD data between RT and 800 °C upon heating and at 350 °C after cooling (open symbol).

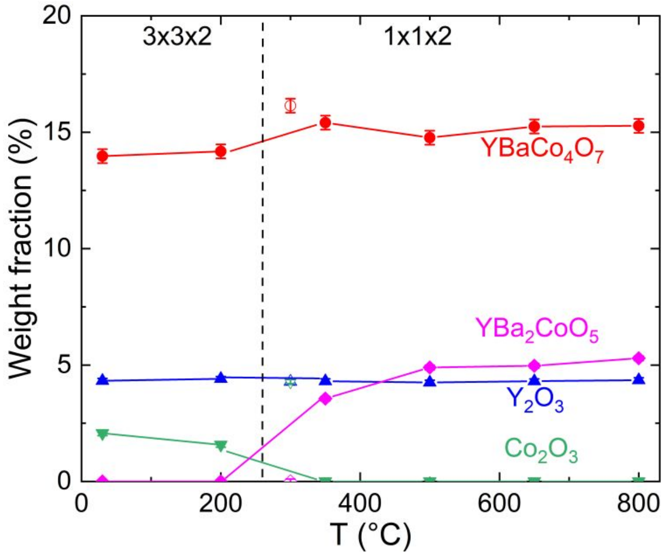

Fig. S5. Temperature evolution of impurity phase weight fractions for the $YBaCo_2O_{6-\delta}$ sample between RT and 800 °C upon heating and after cooling to 300 °C (open symbol) under N_2 flow.

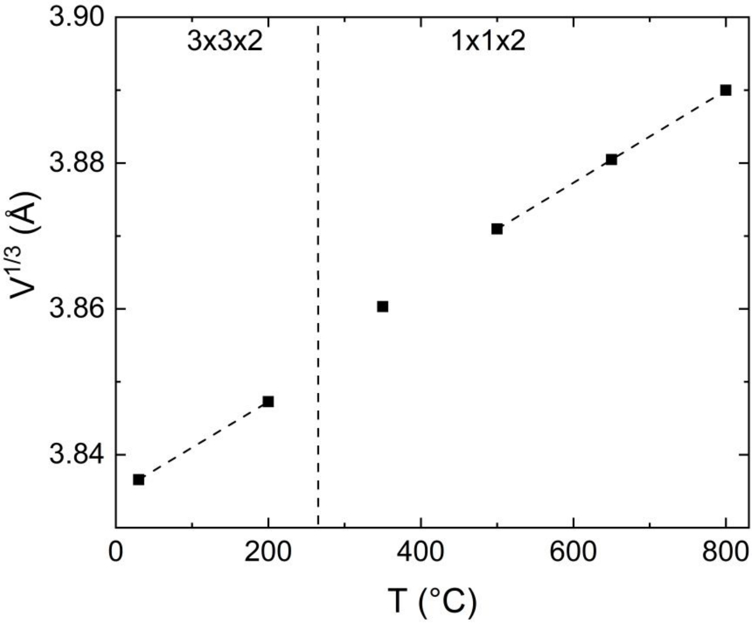

Fig. S6. Linear volume fits of $YBaCo_2O_{6-\delta}$ between RT – 200 °C and 500 – 800 °C (regions of constant δ) to decouple regular thermal expansion from chemical reduction, enabling determination of the linear thermal expansion coefficient, α. Fitted values are given in Table S7.
